# Validation analysis of the novel imaging-based prognostic radiomic signature in patients undergoing primary surgery for advanced high-grade serous ovarian cancer (HGSOC)

**DOI:** 10.1038/s41416-021-01662-w

**Published:** 2021-12-18

**Authors:** Christina Fotopoulou, Andrea Rockall, Haonan Lu, Philippa Lee, Giacomo Avesani, Luca Russo, Federica Petta, Beyhan Ataseven, Kai-Uwe Waltering, Jens Albrecht Koch, William R. Crum, Paula Cunnea, Florian Heitz, Philipp Harter, Eric O. Aboagye, Andreas du Bois, Sonia Prader

**Affiliations:** 1 Department of Surgery and Cancer, Faculty of Medicine, Imperial College London, London, W12 0HS UK; 2 Department of Radiology, Imperial College Healthcare NHS Trust, London, W12 0HS UK; 3 Cancer Imaging Centre, Department of Surgery and Cancer, Faculty of Medicine, Imperial College London, London, W12 0HS UK; 4 Department of Imaging, Oncological Radiotherapy, and Hematology, Fondazione Policlinico Universitario A. Gemelli IRCCS, Rome, Italy; 5 Department of Gynecology and Gynecologic Oncology, Kliniken Essen-Mitte, Henricistr.92, 45136 Essen, Germany; 6 Department of Obstetrics and Gynecology, University Hospital, LMU Munich, München, Germany; 7 Department of Radiology, Kliniken Essen-Mitte, Henricistr.92, 45136 Essen, Germany; 8 Institute of Translational Medicine and Therapeutics (ITMAT), Imperial College, London, UK; 9 Department for Gynecology with the Center for Oncologic Surgery Charité Campus Virchow-Klinikum, Charité—Universitätsmedizin Berlin, corporate member of Freie Universität Berlin, Humboldt-Universität zu Berlin, and Berlin Institute of Health, Berlin, Germany; 10 Department of Obstetrics and Gynecology, Brixen General Hospital, Brixen, Italy; 11 Department of Obstetrics and Gynecology, Innsbruck Medical University, Innsbruck, Austria

**Keywords:** Prognostic markers, Cancer imaging

## Abstract

**Background:**

Predictive models based on radiomics features are novel, highly promising approaches for gynaecological oncology. Here, we wish to assess the prognostic value of the newly discovered Radiomic Prognostic Vector (RPV) in an independent cohort of high-grade serous ovarian cancer (HGSOC) patients, treated within a Centre of Excellence, thus avoiding any bias in treatment quality.

**Methods:**

RPV was calculated using standardised algorithms following segmentation of routine preoperative imaging of patients (*n* = 323) who underwent upfront debulking surgery (01/2011-07/2018). RPV was correlated with operability, survival and adjusted for well-established prognostic factors (age, postoperative residual disease, stage), and compared to previous validation models.

**Results:**

The distribution of low, medium and high RPV scores was 54.2% (*n* = 175), 33.4% (*n* = 108) and 12.4% (*n* = 40) across the cohort, respectively. High RPV scores independently associated with significantly worse progression-free survival (PFS) (HR = 1.69; 95% CI:1.06–2.71; *P* = 0.038), even after adjusting for stage, age, performance status and residual disease. Moreover, lower RPV was significantly associated with total macroscopic tumour clearance (OR = 2.02; 95% CI:1.56–2.62; *P* = 0.00647).

**Conclusions:**

RPV was validated to independently identify those HGSOC patients who will not be operated tumour-free in an optimal setting, and those who will relapse early despite complete tumour clearance upfront. Further prospective, multicentre trials with a translational aspect are warranted for the incorporation of this radiomics approach into clinical routine.

## Background

Surgical and systemic treatment of high-grade serous epithelial ovarian cancer (HGSOC) has experienced unprecedented advances over the last two decades. Through refinement of cytoreductive techniques, the introduction of novel targeted agents and the implementation of genetic and tumour profiling studies, our therapeutic strategies have evolved towards a maximal effort approach across multiple levels that has led to a significant improvement in patient survival [1]. Still, reliable prognostic and predictive biomarkers that would allow the development of more personalised treatment pathways for patients are lacking [2]. The concept of radiomics analysis is a promising novel approach in that direction. Radiomic algorithms based on high-dimensional quantitative features extracted from conventional imaging modalities have been demonstrated to represent a non-invasive platform to quantify tumour heterogeneity and determine prognostic signatures for operability and relapse [3–9].

It is known that despite obtaining total macroscopic tumour clearance at maximal effort upfront debulking surgery, ~25% of patients will relapse early, already in the first postoperative year [10–12]. In an effort to be able to identify those patients with less favourable outcomes, we have recently discovered a novel radiomic signature of tumour phenotype and prognosis, derived from the segmentation of conventional preoperative CT imaging [13]. Using machine-learning and radiomic methods, we derived a non-invasive summary-statistic of the primary ovarian tumour, based on four descriptors, named Radiomic Prognostic Vector (RPV), which reliably identified those patients who would relapse early and have a median overall survival of less than 2 years from initial diagnosis. This novel RPV biomarker was validated in two multicentre independent cohorts: the Hammersmith Hospital, Imperial College Healthcare NHS Trust London (HH), and patients from the Cancer Genome Atlas (TCGA) project [13]. In this study, we wished to evaluate the applicability of our novel radiomics biomarker in an independent cohort of patients, operated within an ESGO (European Society of Gynaecological Oncology) certified Ovarian Cancer Centre of Excellence, the Department of Gynaecologic Oncology, Kliniken Essen-Mitte (KEM). To strengthen the basis for future clinical implementation, data were additionally correlated with our previous findings from the HH cohort.

## Methods

### Patients’ selection and treatment modalities

Retrospective cohort study design with ethical approval for retrospective analysis of human data was obtained under the Hammersmith and Queen Charlotte’s & Chelsea Research Ethics Committee approval 05/QO406/178 and the Kliniken Essen-Mitte Research Ethics Committee approval and informed consent was waived. The study was performed in accordance with the ethical standards of the institutional and/or national research committee and with the principles of the 1964 Declaration of Helsinki and its later amendments or comparable ethical standards. All consecutive HGSOC patients who underwent primary debulking surgery in KEM between January 2011 and July 2018 were screened for the present analysis. Patients were eligible for inclusion if they were confirmed to have HGSOC histology and an evaluable portal venous CT through the primary ovarian tumour mass prior to undergoing upfront cytoreductive surgery, i.e. before any systemic chemotherapy. Any suspicious bulky visible lesions (≥1 cm) from the CT scan were included in the segmentation process. Patients were not eligible if no primary tumour mass was visible due to the initial absence of adnexal mass or due to surgical excision or if the patient has undergone preoperative chemotherapy (see consort diagram in Supplementary Fig. 1).

Patient demographics, surgical and tumour related data were retrieved from a prospectively populated database in the KEM centre; as opposed to the retrospective data retrieval from medical notes in the HH cohort, as described elsewhere [13]. Progression-free survival (PFS) and overall survival (OS) were defined as the time from the date of surgery to the date of first relapse or death, respectively. Staging was defined according to FIGO-criteria for ovarian epithelial carcinoma [14]. Since FIGO staging criteria changed during the observation time, FIGO stage was adapted to 2014 criteria and applied to patients diagnosed before then. Patients were divided into those with total macroscopic tumour clearance (tumour-free; TF) versus those with any macroscopic postoperative residual disease (i.e. >0 mm; non-tumour-free; NTF).

As in the RPV discovery study [13], all operations were performed within a maximal effort setting aiming to achieve total macroscopic tumour clearance as defined by the surgical quality assurance criteria of the ESGO [15] and AGO guidelines (https://www.ago-online.de/en/leitlinien-empfehlungen/leitlinien-empfehlungen/kommission-ovar). The surgical standards, approach and procedures followed at KEM have been extensively described previously [16–18]. All patients were to subsequently receive platinum-based combination chemotherapy, unless contraindications applied such as poor performance status. Maintenance regimens and availability of clinical trials differed between the KEM and the primary HH study setting, mainly due to funding and licensing differences between the European Medical Agency (EMA) and the UK National Institute for Health and Care Excellence (NICE UK).

Oncologic follow-up in KEM was performed according to the German follow-up recommendations, mainly symptom-guided and based on clinical and ultrasonographic examination in combination with CA-125 measurement in the majority of patients; initially 3-monthly for the first 3 years and then 6-monthly. A CT or MRI scan was performed if the above examinations revealed any pathology. Isolated CA-125 increase was not regarded as a recurrence. For the HH cohort, follow-up patterns for patient care were similar. Patients were routinely evaluated at the end of their treatment for evidence of disease recurrence. Clinical examination and CA-125 assessment (if the preoperative value was elevated) were performed every 3 months for the first 2 years and then 6-monthly. Even though a CT/MRI scan was ordered if the above examinations revealed any pathology, no routine ultrasonographic examinations were performed at follow-up in asymptomatic patients. As in KEM, an isolated CA-125 increase was not regarded as a recurrence.

### Description of the radiomic signature and imaging segmentation

Radiomics quantifies mesoscopic tumour phenotype from anatomic or functional images by defining tumour spatial complexity, including first and higher-order statistics, fractal and shape features, generating disease features not appreciated by the naked eye [13]. The development of our radiomic signature, the Radiomic Prognostic Vector (RPV), is described elsewhere and we refer to that source for a more detailed description [13]. The radiomic image features were divided into the following groups: (1) shape and size features, relating to the shape of the tumour; (2) first-order statistics, quantifying tumour voxel intensity characteristics; (3) second-order statistics consisting of textural features which quantify different measures of three-dimensional intra-tumoural heterogeneity and (4) wavelet features calculating the features in Group 2 and 3 after performing wavelet decompositions of the original image using high-pass or low-pass filters from the coiflet 1 family of wavelets. All feature algorithms were implemented within MATLAB [13].

As patients were referred to the cancer centre from a network of cancer units, contrast-enhanced CT scans were acquired at multiple institutions using different manufacturers and different imaging protocols. All anonymised images and data were transferred electronically, and all evaluable primary ovarian tumour masses were segmented separately by trained radiology registrars (PL, FP and LR) using ITK snap (Version 3.2, 2015) and then all segmentations were checked in consensus with two experienced radiologists especially dedicated and trained in ovarian cancer imaging and who took part in the original study (GA and AR). Moreover, although the CT scans were performed by different radiology teams in the two centres for clinical care, the segmentation of scans and generation of the RPV scores in the KEM cohort was performed by the same team as the initial introductory study on the HH patients, to reduce possible bias.

**Fig. 1:**
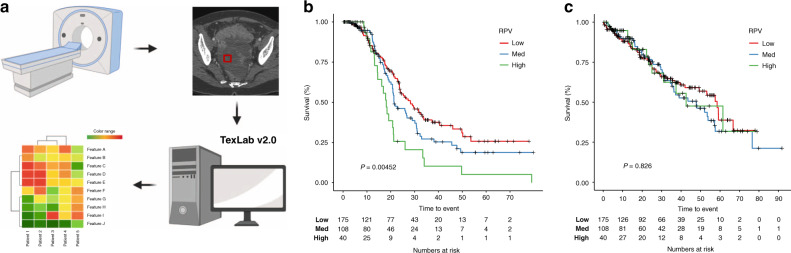
Radiomic prognostic vector (RPV) is associated with progression-free survival in the KEM cohort. **a** Workflow of RPV generation. Patients undergo a CT scan, tumours on the CT scan are segmented and 657 radiomics features are derived using TextLab v2.0 software. RPV score is generated from the derived radiomic features based on previously defined algorithms. Kaplan–Meier analysis showing the association between RPV and **b** progression-free survival or **c** overall survival in the KEM cohort. *P* values are given by log-rank test.

As described previously, the entire primary adnexal mass volume (both cystic and solid components) was included in the analysis [13]. If both adnexa were involved, then both were included in the analysis, either as two separate segmentations or as a single segmentation if the mass was confluent. The segmentations only included tissue that was considered highly likely to be cancer by the expert reader. Areas of doubt on CT were not included in any segmentations. In summary, inclusion criteria related to the CT images were as follows: primary adnexal mass visible, portal venous phase CT through the adnexal mass, no previous surgical or medical treatment for ovarian cancer. Exclusion criteria related to images were: non-contrast or arterial phase CT with no portal venous phase, adnexal mass not included on CT, previous surgery for resection of an adnexal mass, neoadjuvant chemotherapy, the significant artefact for example from metal prostheses that precluded meaningful segmentation of adnexal mass. An outline of the study workflow is shown in Fig. 1a.

The software used was the in-house developed texture analysis package (TextLAB 2.0) developed in MATLAB 2015b (Mathworks Inc., Natick, Massachusetts, USA) [19].

### Statistical analysis

R (https://www.R-project.org/) was used for statistical analysis. Kaplan–Meier analysis was performed using ‘ggkm’ function from ‘survival’ and ‘ggkm’ packages, with arguments ‘pval=T, table=T’. RPV low, medium and high scores were defined as previously described [13]. Cox regression analysis was performed using ‘coxph’ function from ‘survival’ package.

We have chosen well-established and recognised clinical prognosticators of HGSOC [1, 10, 11] to be included as categorical variables in the univariate and multivariable regression to assess the prognostic value of our RPV model. These were: FIGO stage, age, postoperative residual disease and ECOG status.

The first two principal components were tested in the unpaired *t* test to compare variations in radiomic data from KEM, HH and TCGA cohorts. ‘prcomp’ and ‘ggbiplot’ function from ‘stats’ and ‘ggbiplot’ packages were used to perform the principal component analysis (PCA). Radiomic data were centred and scaled before used as input. Two-sided, unpaired *t* tests were performed using t.test function from ‘stats’ package, with argument ‘alternative = “two.sided”, paired = FALSE, conf.level = 0.95’.

## Results

### Patient demographics, tumour characteristics and survival data

All patient- and surgery-related characteristics, as well as survival data for KEM (*n* = 323), are presented with the corresponding information from the primary discovery/validation study cohort from HH (*n* = 224) in Table 1 and consort diagram in Supplementary Fig. 1. Almost half of the patients were older than 60 years of age and the vast majority (92.3%) had advanced Stage III or IV disease. There were significant differences between the two centres, in that KEM had a significantly higher rate of Stage-IV patients, and ECOG status was not known in the majority of the HH patients. For almost one-third of the HH patients, no data were available for postoperative residual disease, mainly attributed to the retrospective data retrieval, therefore these patients were not included in the survival analysis specifically related to postoperative residual disease but were included in the overall survival studies. Approximately half of the patients had relapsed within the follow-up time of the present analysis. There was no difference in overall survival (OS) between the two centres for Stage III or IV patients, as well as PFS for Stage-IV patients. The PFS in Stage-III patients was significantly higher for the KEM cohort (*P* = 0.0312, log-rank test; Supplementary Fig. 2).

**Table 1:** Patient demographics from KEM and HH cohorts.

Characteristics		KEM (*n* = 323)	HH (*n* = 224)	*P* value
Age years (%)	<60	160 (49.5)	97 (43.4)	ns
	>60	163 (50.0)	127 (56.7)	
FIGO stage (%)	I	6 (1.9)	9 (4)	<0.0001
	II	14 (4.3)	16 (7.1)	
	III	132 (40.9)	135 (60.3)	
	IV	166 (51.4)	61 (27.2)	
	Unknown	5 (1.5)	3 (1.3)	
ECOG (%)	0	297	11	<0.0001
	1	20	31	
	2	5	18	
	3	1	2	
	Unknown	–	162	
BRCA status	BRCA1/2 mut	99	–	
	Wild-type	73	–	
	Unknown	151	224	
Residual disease (%)	Tumour-free	196 (60.7)	106 (47.3)	ns
	Non-tumour-free	122 (37.8)	47 (21)	
	Unknown	5 (1.5)	71 (31.7)	
RPV	Low	175	147	0.00253
	Medium	108	66	
	High	40	11	
Follow-up (months; median IQR)		34.5 (13.1–54.4)	49.3 (16.5–71.8)	ns
PFS (months; median IQR)	All	23.9 (16.2–49.7)	21.1 (11.7–50.9)	ns
	Stage III	26.60 (17.50–49.80)	19.41 (12.36–41.28)	0.0312
	Stage IV	21.20 (13.30–31.60)	13.54 (8.89–37.25)	ns
OS (months; median IQR)	All	53.2 (24.7–NR)	51.8 (25.5–NR)	ns
	Stage III	59.10 (27.20–NR)	53.15 (25.97–85.08)	ns
	Stage IV	37.40 (19.70–57.50)	33.51 (19.08–53.05)	ns
Relapsed (%)	No	167 (51.7)	96 (42.9)	ns
	Yes	156 (48.3)	108 (48.2)	
	Unknown	–	20 (8.9)	
Deceased (%)	No	211 (65.3)	133 (59.4)	ns
	Yes	112 (34.7)	90 (40.2)	
	Unknown	–	1 (0.4)	

### Prognostic value of RPV

Overall, the distribution of the KEM- patients to low, medium and high RPV scores was 54.2% (*n* = 175), 33.4% (*n* = 108) and 12.4% (*n* = 40), respectively (Table 1). Lower RPV was associated with higher rates of total macroscopic tumour clearance in the KEM cohort (OR = 2.02; 95% CI: 1.56–2.62, *P* = 0.00647). Patients with high RPV had a significantly worse PFS (median PFS = 18.10 months; IQR: 13.20–26.00 months) compared to those with medium or low RPV (median PFS = 25.90 months; IQR: 17.00–50.20, log-rank test *P* = 0.00452); the latter belonging to the most favourable patient subgroup (Fig. 1b). In order to assess RPV through its ability to predict survival or recurrence by specific landmark time points, we have compared patients’ outcome at 1, 2 and 3 years. Even though the 1-year OS and PFS rates were not significantly different between the different RPV groups (*P* = 0.306 and 0.509, respectively), patients with low/med RPV scores were 26% (*P* = 0.02) and 17% (*P* = 0.11) more likely to be free of progression at 2- and 3 years after surgery respectively, compared to patients with high RPV scores.

**Fig. 2:**
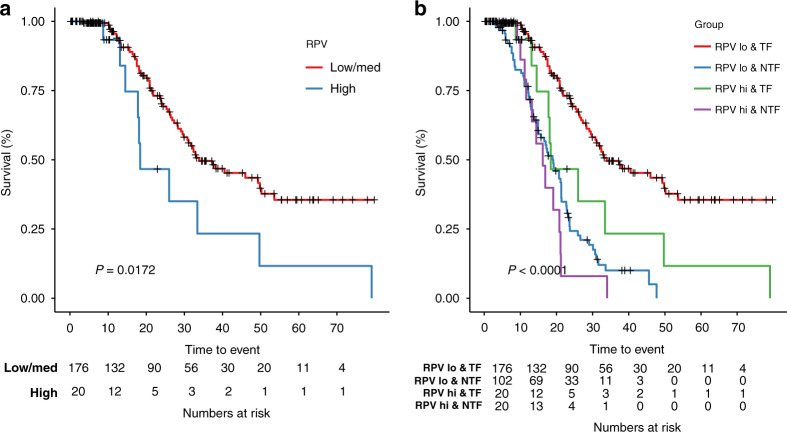
RPV is associated with PFS in patients with postoperative tumour-free status. **a** Kaplan–Meier analysis showing the association between RPV and PFS in the subgroup of patients without postoperative residual disease. **b** Kaplan–Meier analysis showing the association between RPV and postoperative residual disease with PFS in the entire KEM cohort. TF postoperative tumour-free, NTF non-postoperative tumour-free. RPV low and medium subgroups are combined to compare with RPV-high group. *P* values are given by log-rank test.

Patients with lower RPV who were tumour-free following upfront debulking exhibited a better PFS than patients with high RPV (*P* = 0.0172, log-rank test; Fig. 2a), or with any remaining residual disease following surgery (*P* < 0.0001, log-rank test; Fig. 2b).

Univariate analysis identified advanced FIGO stage (as a continuous variable), as well as the presence of any postoperative visible residual disease (versus only microscopic), ECOG and high RPV (versus medium/low), as significant factors for relapse. In multivariable analysis, three factors retained their significant impact on PFS: increasing FIGO stage (hazard ratio (HR) = 1.8; 95% CI: 1.32–2.45; *P* = 0.000191), any postoperative residual disease (HR = 3.00; 95% CI: 2.09–4.32; *P* = 3.16 × 10^−9^) and high RPV versus medium/low (HR = 1.69; 95%CI: 1.06–2.71; *P* = 0.0279); while age (HR = 0.88; 95% CI: 0.62–1.24; *P* = 0.463) and ECOG status (HR = 1.18; 95% CI: 0.722–1.94, *P* = 0.506) did not retain significance in the multivariable analysis (Table 2). No prognostic value of the RPV could be identified for overall survival neither in the entire KEM patients’ cohort nor the tumour-free patient sub-cohort. The known risk factors of FIGO stage and postoperative residual disease [1, 10, 11], were confirmed as significantly affecting overall survival, adding to the validity of our data (Supplementary Table 1). We further examined whether there was any significant association between BRCA status and RPV in the KEM cohort. Information on BRCA mutational status was available for over half of the patients (*n* = 172), however consistent with the RPV discovery study [13], no association was observed between RPV and BRCA status (Supplementary Fig. 3).

**Table 2:** Cox regression analysis for relapse (KEM cohort).

	Univariate		Multivariable	
Feature	HR (95% CI)	*P* value	HR (95% CI)	*P* value
RPV (low/med vs high)	1.94 (1.24–3.04)	**0.00383**	1.69 (1.06–2.71)	**0.0279**
FIGO stage^a^	2.14 (1.59–2.87)	**4.18** **×** **10**^−7^	1.8 (1.32–2.45)	**0.000191**
Residual disease (none vs any)	3.55 (2.54–4.96)	**1.22** **×** **10**^−13^	3.00 (2.09–4.32)	**3.16** **×** **10**^−9^
Age (<60 y vs >60 y)	1.32 (0.964–1.81)	0.0837	0.88 (0.62–1.24)	0.463
ECOG^a^	1.75 (1.12–2.74)	**0.0147**	1.18 (0.722–1.94)	0.506

^a^As continuous variable.

Bold values indicate statistical significance.

### Comparison of radiomic features between the two centres

**Fig. 3:**
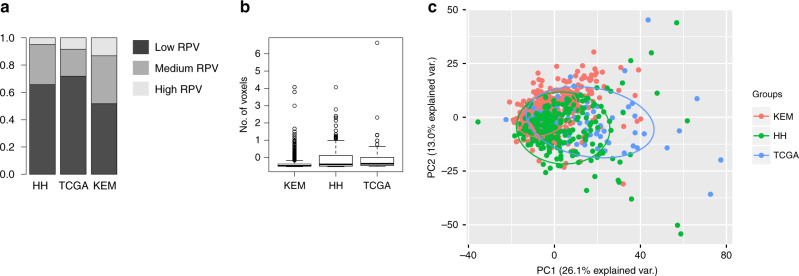
Comparison of radiomics profiles between KEM, TCGA and HH cohorts. **a** Proportion of RPV low, medium and high subgroups in HH, TCGA and KEM cohort; Fishers exact test KEM vs HH (*P* = 0.00253), KEM vs TCGA (*P* = 0.025). **b** Boxplot indicating the volume of the primary tumour segmented from CT scans in HH, TCGA and KEM cohorts; size of the segmented tumours from KEM cohort was significantly smaller than those of HH (*P* = 2.102e-06; *t* test) or TCGA (*P* = 0.00943; *t* test) cohorts. **c** Principal component analysis showing the variation of radiomics profile for cases in HH, TCGA and KEM cohorts. The radiomics profile from HH and TCGA cohorts closely cluster together (*P* > 0.05, *t* test) and are shifted from the radiomics profile for the KEM cohort (*P* < 0.0001, *t* test).

As shown in Fig. 3a, differences exist between the RPV features between the two cohorts which may be attributed to the higher rate of Stage-IV patients in the KEM population. A higher number of patients classified as RPV-high were present in the KEM cohort in comparison to the HH (*P* = 0.00253; Fisher’s exact test) and TCGA (*P* = 0.025; Fisher’s exact test) cohorts. As our previous study showed that RPV is negatively correlated with tumour volume [13], we compared the segmented tumour size among the three cohorts TCGA, HH and KEM (Fig. 3b). Our findings demonstrate that the size of the segmented tumours from the KEM cohort was significantly smaller than those of the HH (*P* = 2.102e-06; *t* test) or TCGA (*P* = 0.00943; *t* test) cohorts, additionally probably contributing to the significantly higher number of KEM patients with RPV-high features.

We performed a principal component analysis to compare the radiomic data structure between KEM, HH and TCGA. As demonstrated in Fig. 3c, the radiomics profile from HH and TCGA cohorts are closely clustered together (*P* > 0.05, *t* test) and shifted from the radiomics profile from the KEM cohort (*P* < 0.0001, *t* test), suggesting a fundamental difference in the radiomic data structure. This may well be attributed to the differences in tumour stage and size between the two cohorts, but we cannot be certain about the exact reasons nor the magnitude of each factor. This would warrant exploring in any future prospective evaluation for RPV.

## Discussion

Our data confirm the promising value of radiomics analysis in patients with HGSOC and additionally enriches the landscape of evidence around radiomic-based approaches in gynaecological cancer overall. We have confirmed the independent prognostic value of the radiomic signature RPV in regards to postoperative residual disease and PFS in HGSOC patients treated within a specialised setting. We demonstrated that those patients with high RPV scores had a significantly worse PFS even after adjustment for other well-established prognostic factors such as FIGO stage, age, performance status and postoperative residual disease. Interestingly, RPV scores did not appear to be influenced by the BRCA status of the patients, confirming our discovery study reports.

By identifying through a robust algorithm, such as the RPV signature, those patients who will relapse early despite our best therapeutic efforts, i.e. primary debulking surgery consolidated by conventional cytotoxic chemotherapy, we can spare them from the unnecessary toxicity that these treatments are potentially associated with, and direct them towards other avenues such as neoadjuvant chemotherapy, early introduction of novel targeted agents, or even omission of cytotoxic chemotherapy and introduction of alternative treatment strategies such as adjuvant drug conjugates or approaches based on molecular profiling, patient-derived organoid, explant or xenograft models.

The fact that the RPV biomarker can be readily calculated by a standardised mathematical model through a segmentation process of routine preoperative patient imaging, automatically translates its potential straightforward implementation into surgical practice for the preoperative stratification of patients towards a more individualised surgical care. Also, the fact that the quality of the preoperative imaging was not standardised and varied between the different centres without having a deleterious effect on the ability to adequately perform the necessary segmentation, the RPV biomarker is likely to be highly generalisable without more specialised training required for its use. This would imply its possible straightforward broader application in different healthcare settings following full validation within a prospective, multicentre trial design.

When assessing the ability of RPV to predict survival or recurrence by specific landmark time points, we could demonstrate, that even though the 1-year OS and PFS rates were not significantly different between the different RPV groups, patients with low/med RPV scores were 26 and 17% more likely to be progression-free at 2- and 3 years after surgery, respectively, in comparison to patients with high RPV. This clearly indicates that even though RPV does not appear to be suited to identify those patients with the highly unfavourable short-term outcome, it is able to do so for more longer-term outcomes.

We have demonstrated a significant association between lower RPV and higher rates of total macroscopic tumour clearance. Nevertheless, prediction of operability was not the aim of our present work. The patients that were included in the present analysis are all operated, so a priori identified from the general cohort as optimal surgical candidates. This means that predictive models of operability were already applied. In addition to that, as both centres were centres of excellence, the rate of patients who had optimal debulking was very high due to accurate patient selection for surgery, resulting in fewer non-tumour-free operated patients. The aim of our work was to identify those patients, who despite the best and maximal therapeutic effort, did not perform well, therefore we can use the RPV to identify those and spare them unnecessary iatrogenic morbidity.

Our results are in accordance with other studies that have similarly identified radiomics nomograms as an effective tool to predict PFS for patients with advanced HGSOC [3–9]. Meier et al. had similarly demonstrated that a higher inter-site cluster variance, as derived from computed tomography by measuring the inter-site texture heterogeneity parameters, was associated with lower PFS and OS in HGSOC patients [20]. Wang et al. were the first to evaluate the radiomics approach in even in more advanced imaging such as PET [8]. They have demonstrated that PET-based radiomic signatures can improve diagnostic accuracy and provide complementary prognostic information compared with the use of clinical factors alone or even combined with radiomic signatures derived from conventional CT, to predict PFS for patients with advanced HGSOC.

To bring it even a step beyond mere prognosticators, Martin-Gonzalez et al. have recently described how current challenges in the treatment of ovarian cancer might be overcome by integrating quantitative radiomic features with the analysis of paired genomic profiles, a combined approach called radiogenomics, to generate virtual biopsies [5]. The association of radiomic signatures with paired molecular profiles were shown to be able to monitor spatiotemporal changes in the heterogeneity of ovarian tumours. The team demonstrated that linking radiomics and biological signatures could potentially improve the clinical management of such a challenging disease by monitoring tumours during the course of therapy and offering additional information for clinical decision making [5]. Our initial RPV study had included integration of parallel genetic, transcriptomic and proteomic profiling of tumours to decipher a biological interpretation of the RPV biomarker, showing activation of stromal phenotype and DNA damage response pathways in RPV-stratified tumours [13].

Nevertheless, despite the encouraging data we were able to generate with regards to the prognostic significance of RPV for OS in the HH and TCGA data, we were unable to validate the RPV for OS in the KEM cohort, raising a question regarding its impact on the prediction of overall clinical and surgical outcome of HGSOC patients. Previously, we have been able to demonstrate even within a multivariable Cox regression model including age, stage and postoperative residual disease, that RPV remained significantly and continuously associated with OS in the TCGA (HR: 4.87, 95% CI:1.67–14.2, *P* = 0.00380) and the HH validation dataset (HR: 7.36, 95% CI: 1.29–41.9, *P* = 0.0245) [13]. These initial data were highly encouraging implying that the addition of RPV would improve the value of the already well-established clinical and surgical prognostic factors, especially since the RPV appeared to carry the strongest impact. The fact that we did not validate this impact now on a larger patient cohort of maximally operated patients generates the dilemma of whether to attempt to repeat our analysis within a prospective trials design or whether to re-evaluate the impact of RPV as a potentially valuable biomarker for overall survival.

We can only hypothesise about the altered impact of the RPV in the different patient cohorts and the lack of reproducibility of its prognostic value regarding OS as opposed to PFS. Since debulking status, stage, age and survival data in the entire cohort were not significantly different between the KEM and HH datasets; the lack of impact of RPV on the OS of the KEM patients cannot be attributed to a different approach in patient selection for surgery between the two centres nor to a presumed superior surgical quality of one centre over the other that would render weak prognostic biomarkers as invaluable within a high-quality treatment setting. The main clinically relevant difference between the two patient cohorts may have been the access to post-chemotherapy maintenance treatment regimens such as antiangiogenetic agents, attributed to the highly significant licensing and funding between EMA and NICE. All Stage III and IV KEM ovarian cancer patients had access to postoperative bevacizumab, as opposed to only Stage IV and non-tumour-free operated Stage-III patients in the HH cohort, outside of clinical trials. As we know that in tumour-free operated Stage-III patients, the addition of bevacizumab has a significant impact only on PFS and not OS [21–23], this may be one of the main reasons for a better PFS in the Stage-III KEM cohort compared to HH.

Still, the aim of this study was not to compare centres and patients’ survival between two different centres, but to evaluate whether the value of RPV could be validated in a second independent centre. For that reason, the data neither were combined in the analysis nor did we assess the value of the factor “centre” as prognosticator.

Various studies have demonstrated the impact of stroma characteristics on the benefit derived from antiangiogenetic treatment which may confound our OS analysis [24–26]. Colon cancer studies for example exploring the independent prognostic value of the tumour–stroma ratio [25, 26] suggest a significantly shorter PFS and OS in stroma-low tumours with the addition of bevacizumab to intravenous oxaliplatin-based chemotherapy, contrary to stroma-high tumours, where a beneficial trend was observed [26]. In high-risk advanced-stage colon cancer, data have shown that even though the stromal organisation itself does not serve as an independent prognostic or predictive parameter; it appears that the combined parameters of stromal organisation and tumour- stroma ratio allow for the identification of those patients who will especially benefit from the addition of bevacizumab to the platinum-based chemotherapy [25]. Therefore, as we know that stromal phenotype and DNA damage response pathways are activated in RPV-stratified tumours [13], it is possible that the higher use of bevacizumab in the KEM- cohort invalidates the OS relationship with a stroma-related prognostic marker such as RPV.

The cystic part of the tumour was not incorporated in the initial development model of the RPV and was therefore equally not extra addressed here. However, the volume/voxel was shown to be correlated with many radiomic features including RPV in our previous study [13], while the prognostic power of RPV was independent of volume/voxel. The use of LASSO method during the development of RPV removed many highly correlated radiomic features, including those features that were all highly correlated with volume/voxel. A future study with a prospective design and a translational aspect may further elucidate these complex interactions of stromal components, antiangiogenetic agents and tumour size and thus establish the true predictive and prognostic value of RPV on OS.

A significant limitation of our study is the lack of correlation of the radiomics features of the KEM cohort with the equivalent biological characteristics of the tumours, as previously performed for the HH and TCGA cohorts due to the lack of histological evaluation of the KEM- specimens [13]. While in the discovery study, an associated molecular analysis of the RPV was possible due to routine banking of tumour tissues post-surgery, it is difficult to obtain further independent large patient cohort retrospective datasets with the same surgical standards with available tissue in sufficient numbers. To address this, we have commenced a prospective collection of CT scans, tumour samples and data in the HH-centre to perform a complete validation of the RPV with molecular analysis, and are developing prospective collaborations for a multicentre translational study. A future prospective study with a translational aspect of a parallel tissue collection to examine differences in tumour cellularity and stroma, fibronectin levels in RPV-high versus low tumours, coupled with targeted multi-omics profiling, will shed more light into understanding the biological background of our findings. Our aim is to determine whether and to what degree and context, stromal content, tumour cellularity, proliferation index, and homologous recombination and DNA damage response pathways, play a role in the biological behaviour of low/medium vs high RPV tumours [27–29].

In conclusion, in this study we demonstrate that patients with high RPV are associated with a 66% probability of a worse PFS regardless of the postoperative residual disease, age, performance status and stage. Although the impact of RPV did not validate for OS, RPV may be deemed valuable, even if only accurate for the prediction of relapse, to direct those patients with a much less favourable profile to alternative or modified treatment strategies. Key tasks towards future clinical use would include validation of the RPV in a large prospective cohort across a multicentre setting that could set the basis for a randomised trial design. Furthermore, we wish to develop a platform for the reliable automation or semi-automation of the segmentation process, which would be a significant advance towards the broader implementation of the RPV algorithm as a biomarker into routine clinical procedures. This would be a step towards a more individualised surgical stratification of patients.

## Supplementary information

Supplementary Figures and Tables

Checklist form

## Data Availability

RPV data and clinical annotations will be available upon publication.
