# Supplementary material for: Validation analysis of the novel imaging-based prognostic radiomic signature in patients undergoing primary surgery for advanced high-grade serous ovarian cancer (HGSOC)
Source: Br J Cancer. 2021 Dec 18;126(7):1047–54. doi: 10.1038/s41416-021-01662-w (PMC8979975; doi:10.1038/s41416-021-01662-w)
Supplement: Supplementary file 1 — Supplementary Figures and Tables [file 41416_2021_1662_MOESM1_ESM.docx]

**Supplementary Figures and Tables**

**Supplementary Figure 1: Consort diagram of study**

**Supplementary Figure 2**

**Supplementary Figure 2. Comparison of PFS and OS in HH and KEM cohorts for stage III and IV patients.** A statistically significant difference in PFS was observed between the KEM and HH cohorts for patients with stage III disease (KEM (median 26.60 months ; IQR: 17.50 - 49.80); HH (median 19.41; IQR 12.36 - 41.28)), with no significant differences observed for stage IV PFS (KEM (median 21.20; IQR: 13.30 - 31.60); HH (median 13.54; IQR 8.89 - 37.25)). No significant differences were observed in OS between each cohort for either stage III (KEM (median 59.10; IQR 27.20;-- NR ); HH (median 53.15; IQR 25.97 - 85.08)) or stage IV disease (KEM (median 37.40; IQR 19.70 - 57.50); HH (median 33.51; IQR 19.08 - 53.05)).

**Supplementary Figure 3:**

**Supplementary Figure 3: RPV scores do not associate with BRCA status.** BRCA status was collected for 172 HGSOC patients from the KEM cohort (BRCA1/2 mut n=99 patients, BRCA wildtype n=73 patients) and associated with corresponding RPV scores. No significant association was observed between RPV and BRCA 1/2 mutation status (p = 0.2).

**Supplementary Table 1: Cox regression analysis for overall survival (KEM cohort)**

|  | **Univariate** | | **Multivariable** | |
| --- | --- | --- | --- | --- |
| **Feature** | **HR (95% CI)** | **p-value** | **HR (95% CI)** | **p-value** |
| RPV (low/med vs high) | 0.967 (0.531-1.76) | 0.912 | 0.697 (0.378-1.29) | 0.249 |
| FIGO Stage* | 2.08 (1.59-2.87) | **6.46x10^-5^** | 1.8 (1.23-2.64) | **0.00249** |
| Residual disease  (none vs any) | 5.08 (3.32-7.76) | **6.12x10^-14^** | 4.17 (2.67-6.52) | **3.78x10^-10^** |
| Age (<60y vs >60y) | 1.79 (1.23-2.6) | **0.00247** | 1.32 (0.88-1.97) | 0.18 |
| ECOG* | 1.86 (1.22-2.84) | **0.00381** | 1.02 (0.62-1.67) | 0.946 |

** as continuous variable*
